# The science of epidemiology and the methods needed for public health assessments: a review of epidemiology textbooks

**DOI:** 10.1186/1471-2458-14-139

**Published:** 2014-02-10

**Authors:** Hebe N Gouda, John W Powles

**Affiliations:** 1 Institute of Public Health, University of Cambridge, Forvie Site, Robinson Way, Cambridge, Cambridgeshire, UK; 2 School of Population Health, Herston Campus, University of Queensland, Brisbane, Queensland, Australia

**Keywords:** Public health, Epidemiological methods, Population health metrics

## Abstract

**Objectives:**

Epidemiology is often described as ‘the science of public health’. Here we aim to assess the extent that epidemiological methods, as covered in contemporary standard textbooks, provide tools that can assess the relative magnitude of public health problems and can be used to help rank and assess public health priorities.

**Study Design:**

Narrative literature review.

**Methods:**

Thirty textbooks were grouped into three categories; pure, extended or applied epidemiology, were reviewed with attention to the ways the discipline is characterised and the nature of the analytical methods described.

**Results:**

Pure texts tend to present a strict hierarchy of methods with those metrics deemed to best serve aetiological inquiry at the top. Extended and applied texts employ broader definitions of epidemiology but in most cases, the metrics described are also those used in aetiological inquiry and may not be optimal for capturing the consequences and social importance of injuries and disease onsets.

**Conclusions:**

The primary scientific purpose of epidemiology, even amongst ‘applied’ textbooks, is aetiological inquiry. Authors do not readily extend to methods suitable for assessing public health problems and priorities.

## Background

The relationship between epidemiology and public health has long been a close one. Traditionally epidemiology is viewed as the main source of analytical tools for public health practitioners and administrators and is frequently described as the ‘basic science of public health’ [1-4]. Many other disciplines are also important to public health. Health economics, sociology and many a number of qualitative approaches to health problems, are a few examples, but epidemiology holds a central role because “of its population focus and quantitative methods” [5]. However, even relationships of long-standing intimacy may not fulfil all the needs of both parties.

In his review of epidemiological textbooks published in 1999, Raj Bhopal noted that these introductory books left readers with no clear tools to approach public health problems with:

Textbooks commonly proclaim the broad applications of epidemiology, particularly as the foundation science of public health,… In my view, textbooks rarely demonstrate clearly how epidemiologic theory is applicable to public health practice. Even those that explicitly combine epidemiology and public health only partly succeed in integrating theory, method and application (p 1164) [6].

Similar critiques have been voiced by others since. In the preface to the textbook, *Statistics in Public Health*, for example, Stroup states that “although numerous texts cover the theory and methods of epidemiology and biostatistics, no single resource has been available to guide analysts in the application of these methods in public health” [7].

A key function of public health is the assessment of population health for the purpose of evaluating policy, interventions and resource allocation [8]. This entails the assessment of public health priorities and for this purpose the consequences of disease onsets becomes an essential extension to an understanding of the causes of those onsets. Time-based metrics which are capable of quantifying the number of years spent in different health states, those that can account for the severity of disability and those that can be used to compare burden between diseases and populations are particularly useful to this end. Metrics like life-years gained (LYG) and disability-adjusted life years (DALY) are valuable metrics designed specifically for the purpose of measuring and directly comparing the burden caused by different public health problems [9].

The question posed here therefore is: Does the scope of epidemiology as manifest in these texts extend to providing techniques helpful in ranking and prioritising health problems? While our investigation is quite focused on the quantitative methods needed to assess and rank public health burdens, broadly related to this are the following questions: How do epidemiologists envisage their purpose in relation to public health? Do epidemiologists acknowledge the utility, for public health purposes, of time-based measures of disease occurrence, and do they include such measures in their set of analytical tools? And, if not, what rationale is given, if any, for concentrating on event-based measures?

To pursue our objective rather than an exhaustive review of all techniques and methods developed by epidemiologists we review a selection of epidemiology textbooks. This is because we consider textbooks here to be a reflection of the dominant discourses within the discipline of epidemiology and to provide a convenient insight into how practitioners envisage the scope of their discipline. Furthermore, textbooks are still the common teaching aid in Masters of Public Health courses. Here we provide an up to date review of epidemiology and public health textbooks with and specifically explore the extent to which standard methodological works by epidemiologists provide all of the analytic tools needed by those assessing public health problems and priorities.

## Methods

### Selection of textbooks

The textbooks selected in the study either focus on epidemiologic methods or present chapters on epidemiologic methods in the context of public health applications. The extent to which the authors are explicitly concerned with the application of epidemiologic methods to public health problems constitutes a spectrum and is categorised here into three groups; ‘pure’ , ‘extended’ and ‘applied’ epidemiology. The groups were defined by the expressed scope of the book and the intended target audience. ‘Pure’ epidemiologic texts are those which aim to address core issues of epidemiologic research to professional epidemiologists or epidemiologists in training. ‘Extended’ texts are those that, in addition to their discussion of core issues, declared intent to extend their coverage to public health applications. ‘Applied’ texts were defined as those where the main declared purpose is to apply epidemiological methods to the assessment of public health problems or priorities.

Our selection of textbooks represented a range of text types, i.e. disease-specific or generic, monographs or edited collections of contributions by multiple authors. This selection was certainly not exhaustive but included some of the more widely used textbooks aimed at graduate students as well as professional epidemiologists and public health practitioners. This was assessed by online searches for textbooks, the observed quantities of library holdings at the Medical Library of the University of Cambridge and by virtue of the text being in its second (or later) edition. We limited the choice to those that were published after 1980 and excluded specialist texts focusing on particular methodologies (e.g. clinical epidemiology, longitudinal methods).

When available, definitions of epidemiology were extracted and categorized as either narrow or broad in scope. Broad definitions are those that include an applied role for epidemiology.

Textbooks were then reviewed with particular attention to the extent that books discussed a range of topics and methods relevant to the public health function of assessing the burden of health problems and prioritization of public health activities, including:

1 - Methods for measuring the levels of health in different populations (or different groups within a larger population), and relatedly, of changes in levels of health over time.

2 - Methods for comparing and ranking the importance to the population’s health of a) different types of diseases and injuries and b) the causes of these different types of diseases and injuries.

For this purpose four criteria were sought:

*Incidence and prevalence as measures of disease occurrence*: Incidence measures the onset of disease while prevalence measures the number of existent cases. In the context of aetiological inquiry, a prevalence measure mixes characteristics associated with the onset of disease with those associated with survival after onset. So for an aetiological study, the inclusion of duration in the metric used ‘muddies the waters’. Here we look at how authors distinguish between these metrics and how they are portrayed.

*Measures of health and disability*: While the onset of disease is preferable for epidemiologists when determining aetiology, the consequences of disease onset is also important for the public health specialist. This information is useful for planning services, policies and/or interventions to reduce morbidity or to assist in the management of disabilities.

*Measures of health and disability designed for comparison*: It is particularly useful from a public health perspective to be able to compare the needs of a population by assessing the burden of different diseases experienced in that population. Such comparisons require metrics that treat different types of diseases and injuries comparably. The DALY is an example of a metric developed with these objectives in mind.

*Lifetable methods*: Lifetables and lifetable methods provide a rich source for time-based health metrics. These include life and health expectancies of a population.

## Results

### Pure texts

Ten texts from each of the three categories were selected (Table 1). Most of the textbooks examined offered a definition of epidemiology in their opening chapters. Amongst the ‘pure texts’ less than half of the authors (4 out of the 10) limit the effective scope of epidemiology to the study of distribution and determinants of disease (Table 2). Rothman and Greenland, for example, explicitly place aetiologic research at the forefront of epidemiology’s task and state that ‘the ultimate goal of most epidemiologic research is the elaboration of causes’ [10]. Of the definitions listed amongst these ‘pure texts’ , those of Szklo *et al.*, [11], Koepsell [12] and Lilienfeld [13] explicitly extend epidemiology’s remit and employ a definition offering a role for epidemiology in the application of epidemiology to the ‘control of health problems’. Two texts, those of Lilienfield [13] and Ahren and Pigeot [14], are distinguished among the pure texts by the breadth of their perspectives on epidemiology – in one case going so far as to describe epidemiology as an ‘eclectic discipline’ made up of methods from a range of other disciplines [13].

**Table 1:** Selected standard epidemiology textbooks, classified as ’pure’, ‘extended’ or ‘applied

**Author**	**Year of publication and edition**	**Title**	**Type**	**Country of authors’ or editors’ origin**
**Pure texts**				
Ahrens, W, and Pigeot, I [14]	2013, 2nd	Handbook of epidemiology	Multi-authored edited compendium	Germany
Gerstman, BB [15]	2003, 2nd	Epidemiology kept simple: an introduction to traditional and modern epidemiology	Monograph	USA
Koepsell, TD, and Weiss, NS [12]	2003	Epidemiologic methods: studying the occurrence of illness	Co-authored monograph	USA
Hennekens, CH, and Buring, JE [16]	1987	Epidemiology in medicine	Edited co-authored monograph	USA
Lilienfeld, DE, and Stolley, PD [13]	1994, 3rd	Foundations of epidemiology	Co-authored monograph	USA
MacMahon, B, and Trichopolous, D [17]	1996, 2nd	Epidemiology: principles and methods	Co-authored monograph	USA
Merrill, RM and Timmreck, TC [18]	2002, 3rd	An introduction to epidemiology	Monograph	USA
Miettinen, O [19]	1985	Theoretical epidemiology: principles of occurrence research in medicine	Monograph	Finland/Canada
Rothman KJ, *et al.*[10]	2008, 3rd	Modern epidemiology	Multi-authored^1^ co-edited volume	USA
Szklo, M, and Nieto, FJ [11]	2012, 3rd	Epidemiology: beyond the basics	Multi-authored monograph	USA
**Extended texts**				
Bonita, R*, et al.*[20]	2006, 2nd	Basic epidemiology	Multi-authored monograph	Australia
Bhopal, R [21]	2008, 2nd	Concepts of epidemiology: an integrated introduction to the ideas, theories, principles and methods of epidemiology	Monograph	UK
Esteve, J, *et al*. [22]	1994	Descriptive epidemiology statistical methods in cancer research	Multi-authored monograph	France
Farmer, R and Lawrenson, R [23]	2004, 5th	Epidemiology and public health medicine	Co-authored monograph	UK
Friis, RH and Sellers, TA [24]	2008, 4th	Epidemiology for public health practice	Co-authored monograph	USA
Gordis, L [25]	2009, 3rd	Epidemiology	Monograph	USA
Moon, G, *et al.*[26]	2000	Epidemiology: an introduction	Multi-authored monograph	UK
Rossignol, A [27]	2005	Principles and practice of epidemiology: an engaged approach	Monograph	USA
Webb, P and Bain, C [28]	2010, 2nd	Essential epidemiology	Multi-authored monograph	Australia
Vetter, N, and Matthews, I [29]	1999	Epidemiology and public health medicine	Co-authored monograph	UK
**Applied texts**				
Brownson, RC, and Petitti, DB [30]	2006, 2nd	Applied epidemiology: theory to practice	Multi-authored co-edited volume	USA
Carr, S *et al.*[31]	2007, 2nd	An introduction to public health and epidemiology	Multi-authored monograph	UK
Detels, R *et al.*[32]	2009, 5th	Oxford textbook of public health: the methods of public health	Multi-authored edited compendium	USA, UK, New Zealand and the Philippines
Donaldson, LJ and Scally, G [33]	2009, 3rd	Donaldson’s essential public health	Co-authored monograph	UK
Gillam, S *et al.*[34]	2007	Essential public health: theory and practice	Multi-authored monograph	UK
Kerr, C *et al.*[35]	1998	Handbook of public health methods	Multi-authored co-edited volume	Australia
Pomerleau, J and McKee, M [36]	2005	Issues in public health	Co-edited volume	UK
Schneider, M-J [37]	2010, 3rd	Introduction to public health	Monograph	USA
Spasoff, RA [38]	1999	Epidemiologic methods for health policy	Monograph	Canada
Tulchinsky, TH and Varavikova, EH [39]	2008, 2nd	The new public health	Co-authored monograph	Israel/Russia

^1^This text is mostly authored by Rothman and Greenland. Only a chapter on Field Epidemiology in Part III and nine chapters in Part IV (Special Topics) are authored by other contributors.

**Table 2:** Definitions of epidemiology employed by all selected standard epidemiology textbooks

**Author**	**Definition of ‘Epidemiology’ (direct quotes unless indicated otherwise)**	**Scope of definition**
Ahrens, W and Pigeot, I [14]	The study of the distribution and determinants of disease frequency (quoting MacMahon [17])	Broad
	And	
	The study of the distribution and determinants of health-related states or events in specified populations, and the application of the study to control of health problems (Quoting Last, [40]) (p 7)	
Gerstman, BB [15]	Epidemiology studies the causes, transmission, incidence, and prevalence of health and disease in human populations. Medical and public health disciplines use epidemiologic study results to solve and control human health problems (p xv)	Broad
	And	
	The study of the distribution and determinants of health-related states or events in specified populations, and the application of this study to the control of health problems (p 3)	
Koepsell, TD and Weiss, NS [12]	In broad terms, epidemiologic research involves describing and interpreting patterns of disease occurrence in populations, in order to generate knowledge that can be used to prevent disease and avoid human suffering (p 17)	Broad
Hennekens, CH and Buring, JE [16]	The study of the distribution and determinants of disease frequency (quoting MacMahon [17])	Narrow
Lilienfeld, DE and Stolley, PD [13]	…epidemiology can be regarded as a sequence of reasoning concerned with biological inferences derived from observations of disease occurrence and related phenomena in human population groups. To this we can add that epidemiology is an integrative, eclectic discipline deriving concepts and methods from other disciplines, such as statistics, sociology, and biology, for the study of disease in a population (p 4)	Broad
MacMahon, B and Trichopolous, D [17]	Epidemiology is the study of the distribution and determinants of disease frequency in human populations (p 1)	Narrow
Merrill, RM and Timmreck, TC [18]	Epidemiology is an investigative method used to detect the cause or source of disease, disorders, syndromes, conditions, or perils that cause pain, injury, illness, disability or death in human populations or groups (p 2)	Narrow
Miettinen, O [19]	…epidemiologic research has been concerned with the frequency of occurrence of illness and related phenomena (states and events) of health and health care (p 4)	Narrow
Rothman KJ and Greenland, S [10]	If the subject of epidemiology inquiry is taken to be the occurrence of disease and other health outcomes, it is reasonable to infer that the ultimate goal of most epidemiological research is the elaboration of causes that can explain patterns of disease occurrence (p 29)	Narrow
Szklo, M and Nieto, FJ [11]	Epidemiology is traditionally defined as the study of the distribution and determinants of health-related states or events in specified populations and the application of this study to control health problems. (p 3)	Broad
Bonita, R *et al.*[20]	Epidemiology is a fundamental science of public health (p 1)	Broad
	The study of the distribution and determinants of health-related states or events in specified populations, and the application of the study to control of health problems (Quoting Last, [40]) (p 2)	
Bhopal, R [21]	Based on what it has done in the last 150 years, epidemiology is the science and practice which describes and explains disease patterns in populations, and puts this knowledge to use to prevent and control disease and improve health (p 14)	Broad
Esteve, J *et al*. [22]	Traditionally, epidemiology is defined as the study of the distribution of diseases over time and place and according to individual characteristics. For the purpose of this book, descriptive epidemiology can be defined by replacing this last term with ‘group characteristics’. This definition encompasses the intended contribution of descriptive epidemiology to etiologic research, as well as emphasising that data known only at a group level are the basis of the discipline (p 2)	Narrow
Farmer, R and Lawrenson, R [23]	Modern methods of epidemiological enquiry were first developed in the course of investigating outbreaks of infectious diseases in the 19^th^ century. In contemporary medical practice the scope and applications of epidemiology have been greatly extended. Similar methods are now used in the development and assessment of preventive programmes and treatments, the assessment of the safety of medicines and in the planning and evaluation of health services. In contrast to clinical medicine, epidemiology involves the study of groups of people (populations) rather than direct study of individuals (p 3)	Broad
Friis, RH and Sellers, TA [24]	Epidemiology is concerned with the distribution and determinants of health and diseases, morbidity, injuries, disability, and mortality in populations. Epidemiologic studies are applied to the control of health problems in populations. The key aspects of this definition are determinants, distribution, population and health phenomena (eg morbidity and mortality) (p 6)	Broad
Gordis, L [25]	The study of the distribution and determinants of health-related states or events in specified populations and the application of this study to control of health problems (quoting Last 1988) (p 3)	Broad
Moon, G *et al.*[26]	Epidemiology is concerned with the distribution and determinants of health and diseases, morbidity, injuries, disability and mortality in populations. It is about the health experiences of human communities (p 2)	Narrow
Rossignol, A [27]	Epidemiology is the foundational science of public health. Much as a yardstick measures length, epidemiologic investigations measure and compare the frequencies of disease, injury, and other health-related events in human populations (p 3)	Narrow
Webb, P *et al*. [28]	Epidemiology, therefore, is about measuring health, identifying the causes of ill-health and intervening to improve health (p 1)	Broad
Vetter, N and Matthews, I [29]	Epidemiology is the study of the distribution and change in diseases (p 3)	Narrow and Broad
	The *purpose* (author’s emphasis) of epidemiology is to identify things in people and their surroundings that affect the occurrence of disease. It forms part of preventive medicine and public health. Epidemiologic methods are also used to assess the effectiveness of new preventive and therapeutic treatments and the impact of different patterns of health care delivery (p 3)	
Brownson RC and Petitti, DB [30]	In our view, applied epidemiology synthesizes and applies the results of etiologic studies to set priorities for intervention; it evaluates public health interventions and policies; it measures the quality and outcome of medical care; and it effectively communicates epidemiologic findings to health professionals and the public. (p ix- preface)	Broad
Carr, S *et al.*[31]	None given	
Detels R *et al.*[32]	Epidemiology is the basic science of public health, because it is the science that describes the relationship of health or disease with other health-related factors in human populations (p 447)	Narrow
	Also uses Last’s definition [40]	
Donaldson, LJ and Scally, G [33]	The epidemiological perspective is a key component in identifying health needs, examining the pattern of disease problems within and between populations, searching for the causes of disease, formulating health promotion and disease prevention strategies, studying the natural history of disease, and planning and evaluating health services. (p 38)	Broad
Gillam, S *et al.*[34]	At the core of epidemiology is the use of quantitative methods to study diseases in human populations and how they may be prevented. Thus epidemiology can be defined as the ‘study of distribution and determinants of health related states and events in the population and the application of this science to control health problems’ (p 24)	Broad
Kerr, C *et al.*[35]	None given	
Pomperleau, J and McKee, M [36]	None given	
Schneider, M-J [37]	Epidemiologic methods are used to investigate causes of diseases, to identify trends in disease occurrence that may influence the need for medical and public health services, and to evaluate the effectiveness of medical and public health interventions (p 51)	Broad
	Epidemiology studies the patterns of disease occurrence in human populations and the factors that influence these patterns (p 52)	
Spasoff, RA [38]	Analytical epidemiology deals with associations between exposures and outcomes, and usually has little concern for the populations in which these epidemiologic phenomena occur. But policy occurs in society, making population directly relevant, so demography and vital statistics are important topics for health policy. (p 32)	Narrow
Tulchinsky, TH and Varavikova, EH [39]	Epidemiology is the study of health events in a population, used to understand disease process and outcome, to determine factors in causation, and to provide direction for medical or public health intervention. The distribution and determinants of health-related states or events in specified populations help to identify potential interventions and priorities to control of health problems (p 114)	Broad

Incidence and prevalence are usually introduced in the first chapters of a textbook and preference for incidence as a measure of disease frequency is often evident in pure texts (5 out of 10 texts presented extended discussions of incidence relative to prevalence) (Table 3). A quote taken from a textbook by Miettinen [14] states clearly the reason one may prefer incidence in aetiologic research:

The study of prevalence, or status distribution, tends to be unattractive from a scientific point of view. The reason is that a parameter of this type is a reflection – a logical composite – of several component parameters, and the development of insight requires studying those components (p 29) [19].

**Table 3:** Characteristics of selected standard epidemiology textbooks: the extent to which authors extend methodological discussions to time-based measures and other public health relevant material

**Author**	**Prevalence/Incidence**	**Measures of health and disability – generic or disease specific**	**Measures of health and disability – designed for comparability**	**Life table methods**
Ahrens, W and Pigeot, I [14]	+/+	Health status measurement and health related quality of life is discussed to some depth	PYLL, DALYs and QALYs all discussed briefly with mention of the issues that arise when measuring health or disease.	A short section included in survival analysis introduces life table methods
Gerstman, BB [15]	+++/+++	N/M	N/M	A full chapter on life tables and another on survival analysis
Koepsell, TD, and Weiss, NS [12]	+/+++	N/M	N/M	A very brief section on survival analysis
Hennekens, CH and Buring, JE [16]	++/+++	N/M	N/M	N/M
Lilienfeld, DE and Stolley, PD [13]	+/+	N/M	N/M	Brief description
MacMahon, B and Trichopolous, D. [17]	++/++	N/M	N/M	Brief section on life tables and survival analysis in ‘Cohort studies’ chapter
Merrill RM and Timmreck, TC [18]	+++/+++	Very brief discussion of activity limitation and ADL scales	N/M	Very brief mention of survivorship studies and life tables
Miettinen, O [19]	+/++	N/M	N/M	N/M
Rothman KJ and Greenland, S [10]	+/+++	N/M	N/M	Life table methods for risk estimation
Szklo, M and Nieto, FJ [11]	+/+++	N/M	N/M	Life table methods discussed in length
Bonita, R *et al.*[20]	+++/+++	Very brief section on measuring disability	Discusses summary measures and one brief section devoted to the DALY	Brief introduction to life expectancy measures
Bhopal, R [21]	+++/+++	N/M	Brief mention of summary measures	N/M
Esteve, J *et al*. [22]	++/+++	N/M	N/M	Full chapter on survival measures. Years of life lost discussed briefly
Farmer, R and Lawrenson, R [23]	+/+	N/M	N/M	Life expectancy mentioned very briefly
Friis, RH, and Sellers, TA [24]	++/++	N/M	Very brief discussion of the DALY	Short section on life tables
Gordis, L [25]	+++/+++	N/M	Brief description of QoL and comparison issues	Substantial discussion of life tables and survival analysis
Moon, G *et al.*[26]	+/+	N/M	N/M	N/M
Rossignol, A [27]	++/+++	N/M	N/M	N/M
Webb, P *et al*. [28]	++/+++	N/M	Brief but thorough discussion of DALYS	Brief description of life expectancy, HALE and DALE
Vetter, N and Matthews, I [29]	+/+	Measures of disability and health are introduced	A relatively extensive discussion of the QALY but no mention of the DALY	Brief mention of life expectancy as a measure population health status
Brownson RC and Petitti, DB [20]	N/M	Condition specific, generic and preference measures are discussed briefly	N/M	N/M
Carr, S *et al.*[31]	+/+	N/M	DALY mentioned very briefly	N/M
Detels R *et al.*[32]	N/M	Disease specific measures discussed, a substantial section is devoted to generic health indicators	DALYs are discussed at length in different chapters	Brief but thorough discussion
Donaldson, LJ and Scally, G [33]	+/+	Disease rating scales mentioned but not discussed in any length	QALY discussed. DALY mentioned as simply the opposite of a QALY	Life expectancy and healthy life expectancy introduced
Gillam, S *et al.*[34]	++/++	N/M	DALY mentioned	Life expectancy is discussed. Life tables mentioned briefly.
Kerr, C *et al.*[35]	+/+	Very brief discussion of the measurement of health states	N/M	One (very short) chapter on life tables
Pomperleau, J and McKee, M [36]	N/M	N/M	Full chapter devoted to BOD and summary measures.	Brief introduction to life expectancy and life tables
Schneider, M-J [37]	+/+	N/M	N/M	Life expectancy and life lost discussed very briefly
Spasoff, RA [38]	N/M	N/M	QALY and DALY are discussed briefly	Life tables are discussed at some length. HALE are discussed briefly
Tulchinsky, TH and Varavikova, EH [39]	+/+	N/M	Very brief mention of “qualitative measures of morbidity and mortality” – QALY, DALY and DFLE	Life expectancy discussed briefly

N/M - Not mentioned.

+/+ − indicates the extent that prevalence and incidence are discussed.

BOD - Burden of Disease.

DALE – Disability adjusted life expectancy.

DALY – Disability adjusted life year.

DFLE – Disability free life expectancy.

HALE – Health adjusted life expectancy.

PYLL – Potential years of life lost.

QALY –Quality adjusted life year.

QoL – Quality of life.

The emphasis is placed on measuring onsets of disease (in other words incidence) as the most ‘fundamental’ measure of disease occurrence.

Many of these texts mention life table methods or life expectancy (8 out of 10) and three provide in-depth discussions and instructive descriptions [10,11,15].

### Extended texts

Apart from the textbooks authored by Esteve [22] and Rossignol [27], all of the ‘extended’ textbooks use broad definitions of epidemiology which extend epidemiology’s purpose to the application of its methods to public health and health service evaluation (Table 2).

As with the ‘pure’ texts, the limitations of prevalence with regards to the study of aetiology are stressed in three of these texts (Table 3). Friis et al. [24] and Merrill et al. [18] explicitly state that an objective of epidemiology is to determine the extent of disease found in a community and to “identify where the public health problem is greatest” p5 [18].

The characterisation of prevalence, as a complexly determined measure is therefore reiterated here. One author (Bhopal) comes to the defence of the prevalence measure, however, proclaiming its value in research and in some kinds of aetiological research.

The prevalence rate [sic] is generally preferred as the measure of burden for long-lasting diseases even when these are rare. For health behaviours and other disease risk factors prevalence is the preferred measure (even in studies of disease causation). Prevalence is sometimes perceived in epidemiology as inferior to incidence. It is not. Both measures have inherent weaknesses and strengths, and different value in various circumstances (p 228) [21].

Several of the other books in this category present brief discussions of life table techniques and/or survival analysis (7 out of 10) and there is evidently greater interest among these books in summary measures intended for comparing across populations like the DALY (5 out of 10) but none of the texts give very much attention to these topics. Webb and Bain [28] mention in passing the Years of Potential Life Lost (YPLL) metric while Gordis [25] devotes a slightly more substantial discussion to life tables, survival analysis and the role of YPLL.

### Applied texts

Most ‘applied texts’ employ broad definitions of epidemiology (5 out of the 7 definitions offered) depicting it as a discipline which can be applied to planning and evaluating specific medical interventions or health services more generally; in some cases an explicit role in helping to set priorities is acknowledged [23,26]. Though we have categorised Spasoff’s definition here as narrow it is in fact stated with a tone of criticism towards epidemiology’s disinterest for populations and indicates the need for other disciplines to meet these needs [38].

With a declared interest in covering a broad range of topics relevant to policy-making and public health action, this group of textbooks often present only a limited discussion of basic epidemiologic methods. Many of these textbooks, for example, do not discuss incidence or prevalence at all. Those that do, however, offer more attention to prevalence than seen in either of the other two categories. Gillam *et al.*, [34]*,* as well as Tulchinsky and Varavikova [39], for example, distinguish the usefulness of the measures according to the chronicity of the disease to be measured. Gillam *et al.* state;

“[this] means that for conditions with a long duration (eg diabetes or heart disease) prevalence is a good estimate of the burden of disease but for conditions of short duration (eg influenza) incidence is a better measure” (p 34) [34].

Tulchinsky and Varavikova similarly note that;

“[i]ncidence is more useful for acute conditions, whereas prevalence is more important in measuring chronic disease and assessing the long-term impact of a disease” (p 124) [39].

Where specific methods are concerned, however, textbooks in this category devote little attention to measures of health levels. For example, Spasoff here briefly describes measures of years of life lost: “Policy epidemiology emphasizes different indicators from those most used in aetiological research… Potential years of life lost (PYLL) is often a more useful statistic than the number of deaths” [38], but stops short of discussing health losses from nonfatal conditions (or time spent with disability).

More books in this category refer to measures of morbidity (4 out of 10) and summary measures of health (7 out of 10) (relative to ‘pure’ and ‘extended’ texts). These discussions are typically brief, only introducing terms and concepts rather than providing any thorough guidance on how to employ them. This is true for most but, of course, not all texts. Detels *et al.*[32], for example, offer a thorough discussion of most topics of interest here, while the textbook by Pomperleau and McKee [36] provides a full discussion of summary measures of health. The latter text, as well as that by Bonita *et al*, make special mention of the importance comparability [20]. Finally, Brownson, is one of the few authors who explicitly state a role for epidemiology in priority-setting: “Establishing public health and health care priorities in an era of limited resources is a demanding task. Epidemiologic tools and approaches can make important contributions to priority setting.” (p 14) [20]. Most texts do not explicitly show how.

## Discussion

The evolution of epidemiologic methods and concepts has been driven by the search of causes of human diseases. It is likely that this will remain the driving force of epidemiologists and of epidemiology (p 123) [41].

Public health has always been critically dependent on aetiologic research and the contributions made by epidemiology. It is not our intention here to suggest that the priority epidemiologists tend to place in the scientific programme and the hierarchy of methods is in any way misguided or misplaced. It does not aim to make any broad statements about the utility of epidemiology to public health action. Epidemiology has growing impact in policy through the increasing evidence based policy making, systematic reviews, and the increasingly influential analysis of the social determinants of health and disease.

Rather our purpose is to highlight how aspirations to extend their scope to provide methods to assess public health problems and priorities have resulted in incomplete coverage of the methodological needs of public health. Rarely has this aspiration led to serious engagements with some of the difficult conceptual and methodological issues involved, for example in the comparison of health levels or in the ranking of health problems for prioritisation. Epidemiological textbooks certainly do not *have* to extend to this task but it is noteworthy that so few do.

Even when their stated purpose is to apply epidemiology to the assessment of public health problems and priorities, most of these authors typically fail to acknowledge that such a purpose entails methods to help rank the social importance of disease occurrences. ‘Applied’ texts claim to extend their scope to encompass the various methods designed to compare and rank health states, to evaluate the public’s health and the public’s needs for health services. Mention may be made of alternative time-based measures of disease occurrence but these explorations typically stop short of full descriptions of the methodologies.

What this paper does show is that textbooks that aim to provide a basic comprehension of epidemiology, even applied epidemiology, neglect a crucially important aspect of what constitutes public health. In *Public Health at the Crossroads*, Robert Beaglehole argues: “Measuring the burden of disease has not been a priority for epidemiologists. ….Furthermore, epidemiologists have not developed methods suitable for the widespread assessment of morbidity” [5]. The growing importance of descriptive exercises like the global burden of disease is evidenced by its influence in global health policy and planning and leaves little doubt that measurement tools like the DALY have a significant place in public health research. The absence of this area of research from the reviewed textbooks, however, suggests that epidemiologists do not see it as central to their task.

Epidemiology is of course not the only science to inform public health action. Health economics, in particular, plays a very influential role in comparing the importance of population health problems and the effectiveness of interventions. The QALY is largely an economic measure and is today the most common metric used in health evaluations. Economic methods, however, may not provide a fully satisfactory analysis of population health needs because of the way in which economic theory is based in individual preferences and in subjective concepts of health. This complicates the linkages to concepts of disease based in ‘objective’ science. Epidemiologists and public health specialists should be encouraged to not simply borrow methods from other disciplines but to develop new methods that encompass the appropriate public health perspectives.

An important limitation in this analysis should be noted. We have not here accounted for the year of publication. Measures of health and morbidity, and composite measures like the QALY and DALY have been in development over the last few decades and one would expect that their use would increase with time. We have also limited this review to general epidemiology texts. Today there are numerous fields and subfields which inform public health decisions and epidemiological methods alike. Biostatistics provides ever more sophisticated approaches to quantification while social epidemiology helps us better understand the ‘causes of the causes’ of population health. A review of general textbooks will not reveal the ‘state of the art’ or ‘cutting edge’ methodologies that are currently in development, or even in use. But what it does expose are the methods and theories that have come to be widely accepted within the field [42]. Authors of epidemiology textbooks should afford more attention to the neglected areas of measuring disease burden and the development of morbidity metrics and tools for the prioritisation of population health problems.

## Abbreviations

DALY: Disability-adjusted life year; HALE: Health adjusted life years; NCD: Non-communicable disease; QALY: Quality-adjusted life year; YPLL: Years of potential life lost.

## Competing interest

The authors declare that they have no competing interests.

## Authors’ contributions

HG conducted the review and drafted the manuscript, JP conceived of the idea and helped draft and edit the manuscript. Both authors read and approved the final manuscript.

## Pre-publication history

The pre-publication history for this paper can be accessed here:

http://www.biomedcentral.com/1471-2458/14/139/prepub
